# The Reproductive Agency Scale (RAS-17): development and validation in a cross-sectional study of pregnant Qatari and non-Qatari Arab Women

**DOI:** 10.1186/s12884-020-03205-2

**Published:** 2020-09-01

**Authors:** Kathryn M. Yount, Laurie James-Hawkins, Hanan F. Abdul Rahim

**Affiliations:** 1 Hubert Department of Global Health and Department of Sociology, Emory University, 1518 Clifton Rd. NE, Atlanta, GA 30322 USA; 2 Department of Sociology, University of Essex, Wivenhoe Park, Colchester, CO4 3SQ UK; 3 Department of Public Health, College of Health Sciences, QU Health, Qatar University, P.O.Box 2713, Doha, Qatar

**Keywords:** Factor analysis, measurement invariance, Qatar, Scale validation, women’s empowerment, women’s agency, Perinatal period

## Abstract

**Background:**

Sustainable Development Goal (SDG) 5 prioritizes women’s empowerment and gender equality, alone and as drivers of other SDGs. Efforts to validate universal measures of women’s empowerment have eclipsed efforts to develop refined measures in local contexts and lifecycle stages. Measures of women’s empowerment across the reproductive lifecycle remain limited, including in the Arab Middle East.

**Methods:**

In this sequential, mixed-methods study, we developed and validated the Reproductive Agency Scale 17 (RAS-17) in 684 women having a normal pregnancy and receiving prenatal care at Hamad Medical Corporation in Doha, Qatar. Participants varied in age (19–46 years), trimester, gravidity (M3.3[SD2.1], range 1–14), and parity (M2.1[SD1.5], range 0–7). Using qualitative research and questionnaire reviews, we developed 44 pregnancy-specific and non-pregnancy-specific agency items. We performed exploratory then confirmatory factor analyses (EFA/CFA) in random split-half samples and multiple-group CFA to assess measurement invariance of the scale across Qatari (*n* = 260) and non-Qatari Arab (*n* = 342) women.

**Results:**

Non-Qatari women agreed more strongly than Qatari women that every woman should have university education, and working outside home benefitted women. Qatari women agreed more strongly than non-Qatari women that a woman should be free to sell her property. Qatari women reported more influence than non-Qatari women in decisions about spending their money (M4.6 versus M4.4), food they can eat (M4.4 versus M4.2), and rest during pregnancy (M4.5 versus M4.2). Qatari and non-Qatari women typically reported going most places with permission if accompanied. A 17-item, three-factor model measuring women’s intrinsic agency or awareness of economic rights (5 items) and instrumental agency in decision-making (5 items) and freedom of movement (7 items) had good fit and was partially invariant across groups.

**Conclusions:**

The RAS-17 is a contextual, multidimensional measure of women’s reproductive agency validated in pregnant Qatari and non-Qatari Arab women. This scale integrates pregnancy-specific and non-pregnancy-specific items in dimensions of intrinsic agency and instrumental agency relevant to Arab women of reproductive age. The RAS-17 may be useful to screen for low reproductive agency as a predictor of maternal and perinatal outcomes. The RAS-17 should be validated in other samples to assess its full applicability across the reproductive life cycle.

## Background

### Introduction

Since 2000, maternal mortality has declined globally, although less than expected when the Millennium Development Goals (MDGs) were agreed [1]. Moreover, the burden of maternal morbidity remains high. In 2015, an estimated 27 million morbidity episodes occurred for the five most common direct obstetric complications, including eclampsia, pre-eclampsia, postpartum haemorrhage, puerperal infection, and abortion [2]. These estimates have raised awareness of the many women who survive complications in pregnancy or childbirth with morbidities. As a result, a woman-centred approach to health and empowerment was embedded in the Sustainable Development Goals (SDGs), Global Strategy for Women’s, Children’s and Adolescents’ Health (2016–2030), and framework for the WHO Maternal Morbidity Working Group [1, 3, 4].

A growing body of evidence suggests that women’s empowerment influences other SDGs [5], including outcomes related to women’s sexual and reproductive health (SRH) [6–10]. These relationships, however, may vary across contexts, SRH outcomes, and dimensions of women’s empowerment [10–12]. Women’s empowerment is a multidimensional construct [13–15] capturing the process by which women claim enabling *resources* to enhance their *agency*, or ability to make strategic life choices in a context of social constraints [16]. *Human resources* may entail schooling or skills-based training; *economic resources* may entail work, income, property, or other assets; and *social resources* may entail relational networks that offer connectedness and support. *Intrinsic agency*—akin to the concept of *thin relational autonomy* [17]—involves a consciousness of one’s capabilities, rights, and aspirations. *Instrumental agency* involves strategic action to pursue one’s aspirations; and *collective agency* involves the identification of group goals and joint actions to pursue those goals [18].

The United Nations’ mandate to prioritize women’s empowerment—in its own right [19] and as a driver of other SDGs [20] has mobilized scientific efforts to conceptualize and to validate measures for women’s empowerment across groups [21], countries [18], and time [22]. Recent efforts confirm our capacity to monitor the progress of nations toward advancing women’s empowerment using comparable measures. Still, women’s empowerment may, in some ways, be context-specific [14, 16, 23, 24], involving some variation in relevant dimensions and related measures. In some contexts, for example, a woman’s expanded freedom of movement may reflect instrumental agency because of prevailing gender norms about postnatal confinement [25]. Elsewhere, a woman who is not subject to these gender norms may not be said to have expanded agency in this domain [21]. Efforts to validate *cross-context* measures of empowerment may have eclipsed efforts to develop *contextual* measures in special populations and lifecycle stages, such as during pregnancy and perinatal periods across the reproductive lifecycle [26].

Measures of women’s empowerment—and agency—that may apply across the reproductive life cycle are lacking. The Sexual Pressure Scale [27, 28] and Sexual Assertiveness Scale [29] focus on empowerment/disempowerment with respect to *sexual activity*. The Sexual Relationship Power Scale (SRPS) assesses agency with respect to *HIV risk and condom use in an intimate relationship* [30]. The Reproductive Autonomy^1^ Scale focuses on the ability *to prevent pregnancy*, and two of the four dimensions have weak psychometric properties [31]. The Reproductive Empowerment Scale focuses on women’s ability *to use contraception* [32, 33]. The Pregnancy-Related Empowerment Scale assesses women’s empowerment *during pregnancy* [34] and has been adapted for use in Sub-Saharan Africa [35]. None of these scales measure pregnancy- and non-pregnancy-specific agency and have unclear relevance in classic patriarchal settings, where women’s influence in household decisions and capacity to move freely may affect their choices about pregnancy- and non-pregnancy-related care.

Here, we developed and validated the Reproductive Agency Scale 17 (RAS-17) to measure women’s agency among diverse pregnant women of reproductive age. The RAS-17 is grounded in concepts of intrinsic and instrumental agency [16, 36, 37] and integrates pregnancy- and non-pregnancy-specific items derived from qualitative research and prior surveys of Arab women of reproductive age. We collected primary data from 684 pregnant women attending prenatal visits at the Women’s Hospital of Hamad Medical Corporation (HMC) in Doha, Qatar, a high-income setting in the classic patriarchal belt where maternal mortality is low and the identification and prevention of perinatal morbidity is prioritized [38, 39]. Foreign nationals comprise about 86% of the resident population, and 90% of foreign nationals are in the working ages (15–64 years). About 10% of foreign workers are non-Qatari Arab nationals from Egypt as well as Syria, Sudan, Lebanon, Jordan, and Palestine [40].

We performed exploratory then confirmatory factor analyses (EFA/CFA) in random split-half samples to identify a final scale with adequate measurement properties [41, 42]. We then used multiple-group CFA to assess the scale’s measurement invariance across pregnant Qatari and non-Qatari Arab women varying in age, trimester, gravidity, and parity and living in Qatar [43, 44]. Evidence of cross-group comparability supports the scale’s use with diverse Arab women in Qatar and sending countries. Findings permit comparison of the RAS-17 with agency measures administered to Arab women of reproductive age but lacking pregnancy-specific items. Findings clarify the salience of developing contextual measures of women’s reproductive agency capturing pregnancy- and non-pregnancy-specific experiences that are valid in pregnancy and the perinatal period in diverse women of reproductive age, and that may apply across the reproductive lifecycle.

### Women’s empowerment and definitions of agency

Women’s empowerment entails three interrelated processes: 1) claims on new *resources*, which may 2) enhance *agency*, and thereby, 3) facilitate the *achievement* of desired life goals [16, 45]. Human resources (like schooling and training), economic resources (like work, earnings, and property), and social resources (like peer networks), typically are observable. Agency, however, is a multidimensional construct that involves internal states of being, ways of acting, and ways of acting jointly with others [16, 46]. *Intrinsic agency*—or *power within*—entails an awareness of one’s own rights, confidence in one’s own capabilities, and motivation to pursue self-defined goals and aspirations. *Instrumental agency* involves the *power to* make one’s own strategic life choices, to act to pursue one’s goals, and to affect change in one’s life. Instrumental agency may be enacted, for example, in household decisions, movement in public spaces, and the expression of views that oppose prevailing norms [21, 47, 48]. *Collective agency* represents the development of group goals and joint actions to achieve shared goals [46, 49, 50]. These three types of agency—*intrinsic*, *instrumental*, and *collective*—can arise in all domains of life, including at home, in the labor market, and in informal and formal political spaces. Women’s agency can be restricted by the normative environment or can challenge restrictive, gendered expectations of roles, responsibilities, and rights [46]. These types of agency also can evolve across lifecycle stages.

### Measurement of women’s agency

Despite agreement that women’s agency is multidimensional [16, 51, 52], its measurement has had notable limitations. First, many researchers have used a single question or summative scale, capturing limited dimensions of women’s agency, often related to decision-making, freedom of movement, *or* financial autonomy [7, 53–57]. A consistent, multidimensional measure of women’s agency still is needed. Second, researchers have paid limited attention to context-specificity in measures of women’s agency [24]. A common practice has been to rely on empowerment measures from multi-country surveys administered to women of reproductive age. The Demographic and Health Survey (DHS) [58, 59] and other multi-country surveys [60, 61] have included questions on intrinsic agency, such as women’s attitudes about physical violence against wives. The DHS and other surveys [61] also have included questions on instrumental agency, such as women’s influence in household decisions and, in some regions, women’s freedom of movement. Although the *dimensions* of intrinsic and instrumental agency are widely relevant, the ways in which these forms of agency manifest may vary [18, 51, 61, 62], and salient local aspects of women’s intrinsic and instrumental agency may be missed.

Third, researchers have tended to create scales of agency that separate pregnancy-specific and non-pregnancy-specific experiences, missing changes and fluctuations across the reproductive lifecycle. One practice has been to focus on non-pregnancy-specific, socio-economic measures of agency among all (ever-married) women of reproductive age, typically 15–49 years. While this group of women is important demographically, their experiences of agency (or non-agency) are diverse, and salient reproductive experiences may not be captured. The Pregnancy-Related Empowerment Scale (PRES), developed in the United States [34], has been used to assess whether group prenatal care improves pregnancy-related empowerment in Malawi and Tanzania [35]; however, the scale has not been validated cross-culturally, including in Arab populations, and cannot track women’s agency *outside of pregnancy* because it includes only pregnancy-specific items. The Reproductive Empowerment Scale of the DHS does not align conceptually with dimensions of women’s intrinsic and instrumental agency and focuses only on *pregnancy prevention* [32, 33]. Women’s agency may change across stages of the reproductive lifecycle [7, 63, 64] and may fluctuate, for example, during menstruation, pregnancy, and the perinatal period [25, 65]. Capturing these changes and fluctuations requires a scale that includes *pregnancy*- and *non-pregnancy-specific* items that can apply to women across the reproductive life cycle.

Finally, validations of women’s agency scales have, only recently, tested for *measurement invariance*, or dimensional and statistical comparability across groups [21], countries [18], and time [22]. Women from different Arab countries, for example, may have different levels of intrinsic or instrumental agency *and* different interpretations of agency-related questions [66]. Variability across groups of women in the measurement properties of an agency scale may preclude comparisons of mean group differences in agency using the same scale [21].

### Women’s agency in the Arab Middle East

In the Arab Middle East, qualitative studies have suggested that women’s intrinsic and instrumental agency are salient and variable across contexts [67, 68]. In one study of women receiving micro-credit in Egypt, women with intrinsic agency in mobility—who felt able to leave the home unaccompanied—felt better able to augment their own well-being [67]. Another study of spousal conflict in Egypt concluded that women valued instrumental agency—or influence in how household earnings were allocated [68]. Among never-married women in Palestine, however, participation in general household decisions was related to experiences of physical and psychological domestic violence [69]. Among agricultural workers in northwest Syria, women have gained some agency without challenging men’s power and privilege in the household [70].

Quantitative studies in the Arab Middle East have assessed the associations of general dimensions of women’s agency with their reproductive health. In Oman, women’s education was a better predictor of contraceptive use than their instrumental agency in household decisions and freedom of movement [71]. In Egypt, women’s decision-making autonomy (a term used by the author) was associated with women’s contraceptive use [72]. In national longitudinal data for Egypt, women’s intrinsic agency (more gender-equitable attitudes) partially mediated a negative relationship between women’s schooling and fertility; however, the mediating role of women’s instrumental (decision-making) agency was more complex [12]. Also, in Egypt, the influences of women’s first birth on their empowerment confirmed the salience of motherhood for women’s empowerment [65]. Finally, in a comparative analysis of the DHS, 23 countries were ranked on *empowerment sources* (such as education and employment) and *empowerment setting* (such as age at marriage) [73]. Jordan and Morocco ranked fourth and twenty-first, respectively, suggesting wide variation in women’s empowerment across Arab countries. In Jordan, the percentage of women making independent decisions ranged from 61.4 for healthcare to 10.5 for major household purchases; whereas, in Morocco, relatively few women reported making independent decisions about purchases for daily household needs (15.4%) and travelling to friends’ homes (8.6%) [73].

Despite these examples, most research on women’s agency has occurred outside the Arab Middle East [21]. Moreover, despite attention to conceptualizing women’s empowerment in the region [74–77], the conceptualization and measurement of women’s *agency* remains limited. To date, no researchers have developed and validated a measure of women’s reproductive agency in the Arab region that is multidimensional, includes pregnancy- and non-pregnancy-specific experiences, and is comparable across a diverse groups of women of reproductive age.

### Study aims and contributions

Here, we have described the sequential, mixed-methods process undertaken to develop and validate the Reproductive Agency Scale 17 (RAS-17) in a diverse sample of pregnant Arab women in Qatar. We have outlined the steps taken 1) to generate an initial pool of pregnancy- and non-pregnancy-specific agency items that draws on theory, narratives from Arab women, and prior surveys, 2) to examine the scale’s measurement properties in pregnant Arab women in Qatar, and 3) to assess comparability of the final scale across pregnant Qatari and non-Qatari Arab women. The RAS-17 warrants validation in other samples and shows promise to screen for aspects of women’s reproductive agency that may predict maternal and perinatal outcomes across the reproductive life cycle in Qatari women and other Arab women in Qatar or sending countries.

## Methods

### Generation of item Pool to measure reproductive agency

The item pool for the Reproductive Agency Scale was identified in the initial phases of a mixed-methods parent study, entitled *Women’s Empowerment and Prenatal Mental Health in Qatar*. Steps to create the item pool included qualitative research, questionnaire review, and pilot testing.

#### Qualitative research, contextual domains of empowerment, and agency-related items

Eligible participants for the qualitative research (more detail available on request) were Qatari women, aged over 18 years, in their second or third trimester of pregnancy, and visiting the Women’s Hospital of Hamad Medical Corporation (HMC) for a prenatal appointment. The Women’s Hospital of HMC is the largest state-owned health care provider, the only tertiary care maternity and neonatal center, and the facility that handles the vast majority of deliveries in Qatar [78]. From May 10, 2016 to August 4, 2016, 26 women completed an in-depth interview before or immediately after their appointment. Trained female qualitative researchers interviewed women in Arabic using a semi-structured guide. Open-ended questions and probes elicited women’s general perceptions of strong and weak women, perceptions of how pregnancy affected women, their ability to access resources and have control over their lives during pregnancy, and experiences related to theoretical domains of empowerment, including: freedom of movement in pregnancy as well as decision-making and experiences of stress in pregnancy. This guide allowed us to explore the concept of women’s empowerment from the perspectives of women, to elicit pregnancy-specific agency items, and to adapt non-pregnancy-specific agency items from existing surveys (see, below).

Qualitative interviews lasted 58 min, on average (range 18–104 min) and were audio recorded with consent or captured via a note taker. Data were reviewed during the fieldwork to identify issues raised by participants that could be explored in subsequent interviews. Data review also enabled the study team to identify when data saturation was reached. Interviews were transcribed verbatim in Arabic, de-identified, and translated into English. Arabic-English bilingual members of the study team checked transcriptions and translations for accuracy.

Data analysis followed a qualitative descriptive design [79], combining inductive and deductive techniques for thematic analysis [80]. Team members read the transcripts multiple times to identify major themes, guided by topics in the interview guide and other themes arising from the data [81]. This approach allowed the team to explore the general question—what do women in Qatar see as “women’s empowerment”?—using women’s own words cumulatively across the interviews without applying prior notions of what empowerment should look like for them [81].

The team then developed inductive codes to capture how women characterized empowered and disempowered women, social resources for empowerment, sources of stress in women’s lives, and women’s experiences with mental health. The data were coded using NVivo software [82]. The team combined line-by-line and thematic coding strategies [83] to capture what women’s empowerment meant for women in Qatar in their own words. The team conducted a second round of coding to ensure accuracy and to capture subthemes identified during the first round of coding. Saturation was reached after the second round of coding, when no new themes were identified in the data. Research team members in Qatar reviewed final codes and analysis with attention to the appropriateness of themes in relation to the experiences and views of Arab women living in Qatar.

Participants defined empowerment in alignment with customary expectations of women in Qatar. They felt that an empowered woman should be able to maintain control over all facets of her life, often including her emotions. Women also saw empowerment as including self-reliance, and the ability to balance multiple roles, such as being a wife and mother. Thus, for many women, pregnancy and motherhood were central to being an empowered woman. At the same time, the gendered expectations attached to control, self-reliance, and gender-role balance were sources of strain for women. This internal conflict between expressed definitions of women’s empowerment and personal experiences of gender-role strain was heightened in pregnancy and motherhood. Based on these themes, the study team adapted statements from the transcripts to include as items capturing women’s *intrinsic reproductive agency* in the survey module.

#### Review of women’s empowerment modules administered in surveys in the Arab region

To complement the qualitative research, the team reviewed modules on women’s empowerment that have been administered to ever-married women of reproductive age (typically 15–49 years) in surveys in the Arab Middle East. Reviewed modules were administered in the Labor Market Panel Surveys [61], Pan-Arab Project for Family Health (PAPFAM) Surveys, Demographic and Health Surveys [84–87], and the team’s own surveys [88] in the region. These reviews confirmed that 1) questions related to intrinsic and instrumental agency have been used in prior surveys, 2) measured aspects of intrinsic agency have included attitudes about gender roles/rights and violence against women, 3) measured aspects of instrumental agency have included decision-making *and* freedom of movement, and 4) pregnancy-specific agency items generally have been lacking. From this review, the team triangulated questions from prior surveys with the qualitative data and adapted questions on instrumental agency related to pregnancy- and non-pregnancy-specific decision-making and freedom of movement that were acceptable to the Qatari context.

#### Questionnaire development, interviewer training, and pilot survey

Following the qualitative research and review of questionnaires, the study team drafted a full questionnaire in English, translated it into Arabic, and back-translated it into English to ensure consistency and accuracy of meaning. Two bilingual members of the Qatar-based study team checked the translation. The questionnaire was programmed in BLAISE to allow for administration through Computer-Assisted Personal Interviews (CAPI). The CAPI format improved the accuracy of data-entry in the field because soft and hard checks were programmed into the questionnaire, and the need for separate data-entry was eliminated.

Twelve interviewers, including a supervisor for each of the evening and morning shifts, with at least a secondary education completed didactic and experiential training on administration of the survey forms. Training included general survey techniques, questionnaire-specific training, and training in use of the CAPI system. Using role play, interviewers practiced administering informed consent and the questionnaire to respondents.

Trained interviews piloted the full questionnaire twice with approximately 70 respondents varying in age, schooling, and socioeconomic status. The first pilot allowed for refinement of the questionnaire and CAPI programming. The second pilot tested a systematic random sampling strategy of interviewing every seventh eligible woman who registered for an appointment to ensure adequate coverage of the women in the waiting room. The study team tested this strategy for the morning and evening shifts. After each pilot, the study team debriefed with interviewers to identify and to address issues, including length of the questionnaire, acceptability of the topics covered, suitability of module and question order, comprehension of question wording, errors in the CAPI programming, and challenges implementing the interval sampling strategy in the study clinics.

#### Final questionnaire and reproductive agency module

The final questionnaire included four modules: one on pre-pregnancy-related enabling human, economic, and social resources, one on intrinsic and instrumental agency in pregnancy-specific and non-pregnancy-specific domains of life, one on mental health (including validated scales for general self-perceived stress, generalized anxiety, and perinatal depression) [39], and one on demographic characteristics and family background. In total, 44 items on women’s agency were retained from the qualitative research, review of Arab questionnaires, and pilot survey (Supplemental Table 1). Nineteen items covered locally salient aspects of *intrinsic reproductive agency*, especially attitudes about gender and women’s economic roles/rights. Twenty-five items covered locally salient aspects of *instrumental reproductive agency*, including 14 items on women’s *influence in family decisions* and 11 items on women’s *freedom of movement*. Pregnancy-specific and non-pregnancy-specific agency items were included in all three item sets.

### Quantitative survey of pregnant women in Qatar

Eligible women for the survey were 18 years or older, Arab nationals, and experiencing a normal pregnancy without complications. Women were recruited and interviewed immediately before or after their prenatal appointment at the Women’s Hospital of Hamad Medical Corporation (HMC). During data collection, supervisors monitored women as they registered for their prenatal appointments and identified eligible women following the inclusion criteria and the piloted random-sampling interval. The supervisor then assigned an available interviewer to approach each selected participant. The interviewer entered into her computer the n^th^ number of the selected participant, and the participant’s disposition was recorded (participation status; accepted or refused). If the woman refused, the interviewer asked five questions to identify reasons for refusal. If the woman consented to be interviewed, the interviewer moved to a private space at a distance from other women in the clinic waiting area to ensure confidentiality and to encourage honest responses. The interviewer then administered the CAPI. The field supervisor provided the Qatar-based study team daily updates on progress and any relevant fieldwork issues. Survey interviews were conducted between January and February 2017.

Six hundred eighty-four of 838 eligible women completed the reproductive agency module (81.6%). On average, women were 30.1 years old (SD = 5.1; range 19–46 years), had married at age 23.6 (SD = 4.2; range 13–43 years) to husbands four and a half years older, had had 3.3 pregnancies (SD = 2.1, range 1–14), had had 2.1 live births (SD = 1.5, range 0–7), were in their second (23%) or third (71%) trimester of pregnancy, and had at least some secondary education or vocational training [39]. Compared to non-Qatari Arab women, Qatari women were younger, married at an earlier age to younger husbands, had had more pregnancies and live births, and had less schooling [39]. Non-Qatari Arab women originated mostly from North Africa (Egypt and Sudan) and the Levant (Jordan, Palestine, Syria, and Palestine).

### Analytic strategy to validate the reproductive agency scale

The study team followed three analytic steps to validate the final Reproductive Agency Scale. First, we performed descriptive analyses to understand distributions and missingness of all initial 44 agency items. Second, in random split-half subsamples, we performed exploratory factor analysis (EFA) starting with all 44 items and then confirmatory factor analysis (CFA) with a final subset of agency items that met model-fit criteria. Third, we performed a sequential, multiple-group CFA to test the measurement invariance of the confirmed factor model across the subsamples of Qatari national women and non-Qatari Arab women living in Qatar.

#### Descriptive analyses

The team assessed distributions and missingness for the 19 attitudinal items designed to capture women’s *intrinsic reproductive agency*. Example pregnancy-specific and non-pregnancy-specific items were “Every woman should have a university education,” “A woman has a weaker personality when she is pregnant,” and “A woman has the right to disagree with her husband in decisions related to having children” (Supplemental Table 1). The original response options were five-point Likert-type scales ranging from *strongly disagree* (=1) to *strongly agree* (=5)*,* with options for *don’t know* and *not relevant*. Non-ordinal response options were coded as missing, and adjacent response categories where collapsed if cell counts were sparse. Items were reverse coded, as needed, so higher scores denoted higher intrinsic agency for all items. Internal reliability of the items was adequate in this sample (alpha = 0.76, *n =* 684).

The team also assessed distributions and missingness for the 25 behavioral items designed to capture women’s *instrumental reproductive agency* in two domains: influence in personal and household decisions (14 items) and freedom of movement (11 items). Example pregnancy-specific and non-pregnancy-specific decision-making items asked each respondent about their extent of influence in “who you married,” “at what age you married,” “when you become pregnant,” “how to spend your money,” and “which doctor you see for your current pregnancy.” The original response options for these items were five-point, Likert-scales ranging from *no influence* (=1) to *I decided myself* (=5), with options for *don’t know* and *not relevant*. Non-ordinal response options were coded as missing, and adjacent response categories where collapsed if cell counts were sparse. Internal reliability of all 14 decision-making items was marginal in this sample (alpha = 0.62, *n* = 684).

Example pregnancy-specific and non-pregnancy-specific freedom-of-movement items asked respondents about their extent of freedom to visit places, such as the hospital, the movies, coffee shops, and a female friend’s house. The original response options for these items were five-point, Likert-type scales ranging from *you are not allowed to go* (=1) to *you go without permission by yourself* (=6), with options for *don’t know* and *not relevant*. Non-ordinal response options were coded as missing, and adjacent response categories where collapsed if cell counts were sparse. The internal reliability of all 11 freedom-of-movement items was adequate in this sample (alpha = 0.84, *n* = 684).

Given the binary or ordinal response options for each item, we estimated polychoric correlations in random split samples (see below) to assess the level of bivariate association between item pairs [89]. These correlation matrices were the basis for exploratory and confirmatory factor analyses.

#### Exploratory and confirmatory factor analyses

Our steps in the factor analysis followed prior work validating scales for agency in women of reproductive age [21, 89–92]. Here, EFA was a useful first step to identify the factor structure for a new reproductive agency scale [90]. Our sample was sufficiently large [41, 42] to perform EFA with one random subsample (*n*_1_ = 342) and then CFA with the second random subsample (*n*_2_ = 342) to confirm the final factor structure identified in the EFA [90].

The team performed EFA to assess the dimensionality and item-factor loadings of the RAS. We estimated sequential one- to five-factor EFA models with all 44 initial items and used oblique rotation, which allowed us to assess how distinctly items loaded onto agency factors when different numbers of factors were specified [93]. We evaluated each model based on theory [21] (e.g., intrinsic and instrumental reproductive agency are distinct, correlated constructs), item-factor loadings, and model fit indices. Items with low loadings (< 0.300) or a significant cross-loading (>|0.300|) on a second factor were removed. Three indices—the Root Mean Square Error of Approximation (RMSEA *close to* 0.06 or less), Comparative Fit Index, (CFI *close to* 0.95 or greater), and Tucker-Lewis Index (TLI *close to* 0.95 or greater)—were used to assess model fit [41, 42]. The one- and two-factor models had poor fit to the data, and the four- and five-factor models either did not coincide with theory or had fewer than the suggested minimum of three items loading per factor (results available on request) [94]. In the three-factor EFA model with all 44 items, 16 *attitudinal* items pertaining to women’s role in personal and household decisions had low loadings or cross-loadings, and were dropped. Seven items pertaining to *behavioural* decision-making with low loadings or cross-loadings on a second factor were dropped. Four items pertaining to *behavioural* freedom of movement with low loadings or cross-loadings on the third factor were dropped. A three-factor, 17-item model was selected as the best fit to the data. Five items pertained to women’s intrinsic reproductive agency or awareness of their economic rights. Six items pertained to instrumental reproductive agency in household decisions related to self and family (3 items), self-care in pregnancy (2 items), and visits to the hospital (1 item). Six items pertained to instrumental reproductive agency in freedom of movement (6 items). We performed a CFA in the other random subsample (*n*_2_ = 342) to test the final, 17-item, three-factor EFA model and used the same criteria to assess model fit.

#### Measurement invariance analysis

Next, we estimated a series of nested, multiple-group confirmatory factor models to assess configural, metric, scalar, and strict measurement invariance of the RAS-17 across Qatari national and non-Qatari Arab women [95]. Evidence of measurement invariance across these groups would suggest that the scale was useful to screen for women with low reproductive agency. To assess configural (or dimensional) invariance, we estimated the three-factor CFA model for each group, but allowed parameter estimates (item-factor loadings or slopes, thresholds or intercepts, and residual variances) to differ across groups. To assess metric invariance, we constrained item-factor loadings to be equal but allowed item thresholds and residual variances to differ across groups. To assess scalar invariance, we constrained item-factor loadings and thresholds to be equal but allowed item residual variances to differ across groups. To assess strict factorial invariance, we constrained item factor loadings, thresholds, and residual variances to be equal across groups. In practice, strict invariance is rarely achieved and does not preclude a determination of measurement invariance [95]. For each model, we used the RMSEA, its 90% confidence interval, the CFI, and TLI to assess model fit. In addition, non-significant differences in the chi-square (*X*^2^) test statistics between sequential, nested models provided evidence of measurement invariance with respect to each additional equality constraint. If invariance was not achieved at any step (e.g., the difference in the chi-squared test statistics was significant), we consulted modification indices, and freed model parameters to determine if partial invariance could be achieved.

## Results

### Descriptive statistics

Table 1 provides descriptive statistics for the 17 items retained in the final, three-factor EFA model. On average, women agreed or strongly agreed with items representing intrinsic reproductive agency, with item-level scores ranging from 3.99 to 4.52 (Table 1). On average, non-Qatari Arab women agreed more strongly than Qatari national women that every woman should have a university education and that working outside the home “strengthens a woman’s personality.” However, Qatari women agreed more strongly than non-Qatari Arab women that a woman should be free to sell her own property (Table 1).

**Table 1:** Distribution of items in the Reproductive Agency Scale 17 (RAS-17), 684 pregnant women 19–46 years attending prenatal appointments at Hamad Medical Corporation Maternity Hospital, Doha, Qatar

Scale items by factor and sub-factor	Qatari *N* = 260			Arab Non-Qatari *N* = 424			All Women *N* = 684				
	*M*	*SD*	(n)	*M*	*SD*	(n)	*M*	*SD*	(N)	*Obs.* Range	*p*
**Intrinsic Reproductive Agency**^a^											
*Awareness of economic rights. Now I’m going read some statements to you. Please tell me how much you agree or disagree with each statement, where 1 is strongly disagree and 5 is strongly agree.*											
Every woman should have a university education; *univedu*	4.37	*0.05*	(260)	4.61	*0.03*	(424)	4.52	*0.03*	(684)	1–5	***
A woman is powerful if she works for pay at a job outside the home; *workpay*	4.12	*0.06*	(260)	4.01	*0.05*	(424)	4.05	*0.04*	(684)	1–5	
A woman should be free to sell her own property; *sellprop*	4.17	*0.05*	(259)	3.99	*0.04*	(424)	4.06	*0.03*	(683)	1–5	**
Financial independence makes a woman strong; *finanind*	4.07	*0.07*	(258)	3.95	*0.05*	(423)	3.99	*0.04*	(681)	1–5	
Independent of money, working outside the home strengthens a woman’s personality; *wkouthm*	3.97	*0.06*	(258)	4.17	*0.04*	(424)	4.10	*0.04*	(682)	1–5	**
**Instrumental Reproductive Agency**^b^											
*Influence in personal and family decisions. How much influence have you had in the following decisions: 1 = no influence, 2 = a little influence, 3 = some influence, 4 = a lot of influence, and 5 = I decided by myself.*											
How to spend your own money; *decmoney*	4.62	*0.07*	(244)	4.39	*0.06*	(388)	4.48	*0.05*	(632)	1–5	*
Whether or not you can leave the house unaccompanied; *dleavehm*	3.76	*0.09*	(204)	3.67	*0.07*	(309)	3.70	*0.06*	(513)	1–5	
What food available in the house you can eat; *dfoodeat*	4.41	*0.07*	(254)	4.21	*0.06*	(419)	4.28	*0.04*	(673)	1–5	*
How much you work during your current pregnancy; *dwrkprg*	4.18	*0.10*	(182)	4.02	*0.09*	(217)	4.10	*0.07*	(399)	1–5	
How much you rest during your current pregnancy; *drestprg*	4.45	*0.06*	(251)	4.18	*0.06*	(397)	4.28	*0.05*	(648)	1–5	**
*Freedom of movement. Under what circumstances are you allowed to go to the following places: 1 = you are not allowed to go, 2 = you do not go, 3 = you go with permission if accompanied, 4 = you go with permission by yourself, 5 = you go without permission if accompanied, and 6 = you go without permission by yourself.*											
	Mode	*%*	(n)	Mode	*%*	(n)	Mode	*%*	(n)	*Obs.* Range	
The hospital; *fomhos*	4	*55.4*	(251)	3	*50.6*	(419)	4	*52.4*	(670)	1–6	
The movies; *fommovie*	3	*60.4*	(235)	3	*56.4*	(388)	3	*58.0*	(623)	1–6	
Restaurants at hotels; *fomrest*	3	*74.3*	(237)	3	*73.0*	(400)	3	*73.5*	(637)	1–6	
Coffee shops; *fomcoffe*	3	*69.0*	(242)	3	*70.0*	(404)	3	*69.7*	(646)	1–6	
The mall; *fommall*	3	*58.8*	(250)	3	*61.5*	(418)	3	*60.5*	(668)	1–6	
A female friend’s house; *fomfriend*	4	*71.0*	(248)	4	*72.5*	(396)	4	*71.9*	(644)	1–6	
Parks or gardens; *fomparks*	3	*71.7*	(251)	3	*73.0*	(418)	3	*72.5*	(669)	1–6	

Notes. Percent missing for *dwrkprg* (42%) was high because not all women in the sample were employed. Missing cases across remaining items ranged 0–10% for Qatari women, 0–8% for non-Qatari women, and 0–9% for all women

^a^Items derived from the qualitative transcripts

^b^Items adapted from existing surveys, based on a comparison with the qualitative data

*p < 0.05, **p < 0.01, ***p < 0.001 for independent samples t-test (two-tailed) comparing Qatari and non-Qatari women

Women also reported that they had a lot of influence in household and personal decisions; however, for most decisions, women did not report making decisions independently, on average. Compared to non-Qatari Arab women, Qatari women reported a higher mean influence in decisions about how to spend their own money (4.62 versus 4.39), what food in the house they can eat (4.41 versus 4.21), and how much they rest during their current pregnancy (4.45 versus 4.18). With respect to women’s freedom of movement, a majority of Qatari and non-Qatari Arab women reported going most places with permission if accompanied; however, a majority of both groups reported going to a female friend’s house with permission unaccompanied (Table 1).

Table 2 presents polychoric correlations for the final 17 agency items. Items that captured intrinsic reproductive agency had pairwise correlations ranging from 0.21 to 0.55. Items that captured instrumental reproductive agency had more variable pairwise correlations.

**Table 2:** Polychoric correlations of items in the Reproductive Agency Scale 17 (RAS-17), 684 pregnant women attending prenatal appointments at Hamad Medical Corporation Maternity Hospital in Doha, Qatar

Variable Name	1	2	3	4	5	6	7	8	9	10	11	12	13	14	15	16	17
(1) univedu	1.00																
(2) workpay	0.37	1.00															
(3) sellprop	0.21	0.33	1.00														
(4) finanind	0.25	0.55	0.45	1.00													
(5) wkouthm	0.33	0.52	0.28	0.54	1.00												
(6) dmoney	−0.09	−0.01	−0.13	−0.05	−0.04	1.00											
(7) dleavehm	0.08	0.16	0.18	0.14	0.12	0.02	1.00										
(8) dfoodeat	0.15	0.15	0.14	0.15	0.17	− 0.05	0.17	1.00									
(9) dwrkprg	0.08	0.29	0.22	0.28	0.24	− 0.06	0.17	0.32	1.00								
(10) drestprg	0.04	−0.17	−0.06	−0.07	− 0.07	− 0.05	− 0.04	−0.24	− 0.27	1.00							
(11) fomhosp	0.07	0.07	0.10	0.03	0.06	−0.04	0.10	0.02	0.01	−0.00	1.00						
(12) fommovie	0.16	0.08	0.17	0.18	0.15	−0.11	0.18	0.12	0.23	0.02	−0.05	1.00					
(13) fomrest	0.06	0.15	0.06	0.15	0.07	0.04	0.04	0.12	0.05	−0.12	−0.11	0.01	1.00				
(14) fomcoffe	0.12	−0.02	−0.01	− 0.03	0.12	− 0.06	−0.03	0.06	−0.00	− 0.01	0.09	0.09	−0.26	1.00			
(15) fommall	0.03	0.01	−0.06	−0.01	0.03	−0.06	0.01	−0.19	− 0.16	0.20	− 0.01	0.06	− 0.07	−0.09	1.00		
(16) fomfriend	0.10	0.19	0.15	0.21	0.16	0.01	0.06	0.12	0.24	−0.17	−0.08	0.15	0.17	−0.01	−0.03	1.00	
(17) fomparks	0.07	0.07	0.10	0.07	−0.00	− 0.12	0.08	− 0.02	−0.00	0.01	−0.01	0.03	0.00	−0.15	0.13	0.09	1.00

### Exploratory and confirmatory factor analyses

In the final, 17-item, three-factor EFA and CFA models, all items loaded significantly on their respective factors at or above 0.30 (Table 3). Loadings for the five intrinsic-agency items ranged from 0.40 to 0.81 in the EFA model and 0.44 to 0.84 in the CFA model. Loadings for the five instrumental-agency in decision-making items ranged from 0.32 to 0.88 in the EFA model and 0.47 to 0.96 in the CFA model. Factor loadings for the seven instrumental-agency freedom-of-movement items ranged from 0.47 to 0.84 in the EFA model and 0.50 to 0.83 in the CFA model. The EFA and CFA models had good fit to the data (EFA: RMSEA = 0.06; CFI = 0.95, TLI = 0.93; CFA: RMSEA = 0.06; CFI = 0.95, TLI = 0.94).

**Table 3:** Final factor loadings and fit statistics for exploratory factor analysis (EFA, *N*_*1*_ = 342) and confirmatory factor analysis (CFA, *N*_*2*_ = 342) of Reproductive Agency Scale 17 (RAS-17) scale items in split-half samples of pregnant women attending prenatal appointments at Hamad Medical Corporation Maternity Hospital in Doha, Qatar

(#) Scale item by factor; variable name	EFA Factor Loadings			CFA Factor Loadings		
	1	2	3	1	2	3
*Factor 1: Intrinsic reproductive agency (awareness of economic rights)*						
(1) Every woman should have a university education; univedu	.40			.44		
(2) A woman is powerful if she works outside the home; workpay	.76			.67		
(3) A woman should be free to sell her own property; sellprop	.58			.63		
(4) Financial independence makes a woman strong; finanind	.81			.84		
(5) Independent of money, working outside the home strengthens a woman’s personality; wkouthm	.70			.65		
*Factor 2: Instrumental reproductive agency (influence in personal and family decisions)*						
(6) Decision to rest during pregnancy; drestprg		.80			.67	
(7) Decision about how to spend money; dmoney		.61			.62	
(8) Decision to leave house; dleavehm		.32			.47	
(9) Decision about what food can eat; dfoodeat		.50			.53	
(10) Decision about how much work during pregnancy; dwrkpreg		.88			.96	
*Factor 3: Instrumental reproductive agency (freedom of movement)*						
(11) Hospital; fomhosp)			.47			.50
(12) The movies; fommovie			.62			.71
(13) Restaurants at hotels; fomrest			.84			.82
(14) Coffee shops; fomcoffe			.83			.83
(15) The mall; fommall			.76			.71
(16) A female friend’s house; fomfriend			.60			.59
(17) Parks or gardens; fomparks			.66			.69
*Fit Statistics*						
χ^2^			207.85			238.27
RMSEA			.06			.06
CFI			.95			.95
TLI			.93			.95

Notes. CFA uses robust weighted least squares (WLSMV) estimation, probit link

### Measurement invariance of the reproductive agency scale by nationality

Based on the final CFA model, we estimated a baseline, three-factor multiple-group confirmatory model by nationality, without constraining parameter estimates to be equal across groups (Table 4, Model 1). The overall model fit was acceptable (RMSEA = 0.07; CFI = 0.94, TLI = 0.93), suggesting configural invariance. In Model 2, we constrained factor loadings to be equal across groups. Overall, Model 2 showed acceptable fit to the data (RMSEA = 0.06; CFI = 0.94, TLI = 0.93); however, the difference in the chi-squared test statistic between Model 1 and Model 2 was significant. Modification indices suggested that the item with the strongest difference in the loading between groups was “freedom of movement: going to the movies.” A partial metric invariance model that freed the factor loading for this item across groups (Model 3) had an acceptable fit to the data (RMSEA = 0.06; CFI = 0.94, TLI = 0.94), and the difference in the chi-squared test statistics between Model 1 and Model 3 was not significant (*X*^2^ = 20.80, *p* = 0.08).

**Table 4:** Fit indices for multiple-group measurement invariance tests of the Reproductive Agency Scale 17 (RAS-17) across Qatari and non-Qatari Arab pregnant women (*N*=684) attending prenatal appointments at Hamad Medical Corporation Maternity Hospital in Doha, Qatar

Model	Free params	χ^2^ (df)	RMSEA	90% CI RMSEA	CFI	TLI	∆χ^2^	*p*-value χ^2^
M1: Configural invariance	116	577.88 (232)	.07	.06–.07	.94	.93	–	–
M2: Full metric invariance	102	594.36(246)	.06	.06–.07	.94	.93	–	–
M3: Partial metric invariance (removed: *fommovie*)	103	575.76 (242)	.06	.06–.07	.94	.94	20.80	.08
M4: Full scalar invariance	68	679.41 (280)	.07	.06–.07	.93	.93	–	–
M5: Partial scalar invariance (thresholds freed: *univedu, wkouthm, dleavehm*)	73	616.95 (275)	.06	.05–.07	.94	.94	47.65	.02^*^
M6: Full strict invariance	90	603.55 (258)	.06	.06–.07	.94	.94	–	–
M7: Partial strict invariance (residual freed: *wkouthm*)	74	610.34 (274)	.06	.05–.07	.94	.94	20.86	.18

Note: Params = parameter. Statistics ∆χ^2^ and *p*-valueχ^2^ are associated with difference testing. CFA uses WLSMV estimation, probit link. Mplus handles missingness with multiple imputation using Bayesian analysis

^*^p < .05

Next, we compared the partial metric invariance model (Model 3) to one that constrained factor loadings and thresholds to be equal across groups (Model 4), although the item freed in Model 3 remained unconstrained (Table 4). Model 4 had an adequate fit to the data (RMSEA = 0.07; CFI = 0.93, TLI = 0.93), but overall, was significantly different from Model 3. Examination of modification indices suggested freeing the thresholds for three items: “Every women should have a university education”, “Independent of money, working outside the home strengthens a woman’s personality”, and “I decided how much to work during pregnancy.” We estimated an additional model (Model 5) freeing these thresholds; however, Model 5 remained significantly different from Model 3 (*X*^2^ = 47.65, *p* = 0.02). We, then, compared Model 5 to one in which we constrained item factor loadings, thresholds, and residual variances to be equal across groups, with the exception of items that were freed to vary in prior models. Model 6 (the strict invariance model) was significantly different from Model 5 (the partial scalar invariant model). We consulted modification indices, and as a result, freed the residual variance for the item “Independent of money, working outside the home strengthens a woman’s personality,” in Model 7. We then compared Model 7 to Model 5 and found no significant difference (*X*^2^ = 20.86, *p* = 0.18).

## Discussion

### Summary and interpretation

In this study, we used a sequential, mixed-methods approach to develop the Reproductive Agency Scale 17 (RAS-17) in pregnant Qatari and non-Qatari Arab women of reproductive age. Our approach leveraged existing theory, qualitative narratives from women about empowerment within and outside of pregnancy, a review of empowerment modules administered in national surveys to ever-married Arab women of reproductive age, and a psychometric assessment of scale items that captured pregnancy-specific and non-pregnancy-specific agency. The analysis confirmed our capacity to measure intrinsic and instrumental dimensions of reproductive agency in a sample of pregnant Arab women that varied in age, gravidity, parity, and trimester and resided in Qatar, a historically patriarchal, rapidly changing Arab country. The RAS-17 aligns well with theories of women’s empowerment [16], which identify women’s intrinsic agency, instrumental agency in decision-making, and instrumental agency in mobility as distinct, correlated constructs.

Our measure of women’s intrinsic reproductive agency as awareness of their economic rights differed from prior scales, which captured intrinsic agency as non-justification of intimate partner violence (IPV) [18, 21]. The local relevance of women’s awareness of their economic rights, and the importance of this construct in the SDGs [96], suggests that our measure of intrinsic agency should be considered in scales of women’s general and reproductive agency. Notably, we did not ask questions regarding women’s attitudes about IPV; therefore, future scales may consider multiple, correlated dimensions of women’s intrinsic reproductive agency.

Our measure of women’s reproductive agency also aligned well with non-pregnancy-specific measures of women’s instrumental agency in household decisions and freedom of movement, which have appeared in surveys of ever-married women of reproductive age in the Arab Middle East [21] and elsewhere [97]. An innovation of the RAS-17 was that we contextualized these item sets to include salient, pregnancy-specific aspects of decision-making and freedom of movement for Arab women in Qatar. For example, in the initial item pool, we included an item on women’s decision-making about the timing of pregnancy, which appears in the Reproductive Autonomy Scale [31]. However, in the non-Western setting of Qatar, the salient (and psychometrically sound) items for reproductive decision-making concerned women’s ability to influence decisions about how much to work and how much to rest during pregnancy.

Finally, qualitative interviews with pregnant women identified motherhood as a salient aspect of women’s empowerment. This qualitative finding corroborates research in other Arab countries [65], and was reflected in three retained items, on women’s influence in decisions about self-care in pregnancy and freedom of movement to the hospital. The even greater salience of motherhood in the qualitative data suggests the importance of mixed-methods research to understand fully women’s perspectives on and experiences of empowerment.

### Limitations and strengths

Certain limitations of this analysis are notable. First, pre-conception women, pregnant women less than 18 years, and post-partum women were not included in the sample. Our study, therefore, pertains to pregnant women of reproductive age; however, the RAS-17 may have applicability across the reproductive lifecycle. To assess this possibility, this validation study should be repeated in other samples that capture the full range of the reproductive lifecycle. Second, the data were self-reported responses in a cross-sectional survey conducted in a clinic-based sample in a single country. Still, the intensive focus of this research on pregnant women in Qatar offered novel insights about an understudied, and potentially vulnerable, population of women. Moreover, participants included women who differed in Arab nationality, age, age at marriage, trimester, gravidity, and parity, and because most women in Qatar receive prenatal and delivery care at HMC, the sample neared representativeness of pregnant Arab women across the reproductive life cycle in Qatar. The use of fixed-interval sampling also helped to ensure representativeness of the diverse population of clients attending the study clinic. The field team was able to achieve a high response rate (81.6%), demonstrating the feasibility of recruiting pregnant women before or immediately after their clinic appointments.

Finally, our team did not use techniques, such as item-response theory methods, which are useful to assess the precision of items and scales along the underlying continua of hypothesized latent constructs [98]. While these methods can be useful, they can be computationally demanding and complex to interpret for multidimensional constructs and item sets with ordinal response options. Our sequential, mixed-methods approach relied on theory, qualitative research, a review of empowerment modules in national surveys of ever-married Arab women of reproductive age, and a rigorous psychometric assessment involving EFA, CFA, and multiple-group CFA to assess the measurement properties of the RAS-17 across Qatari nationals and non-Qatari Arab nationals. The RAS-17 aligns well with multidimensional theories of agency, has good measurement properties, and has good comparability across diverse pregnant Arab women.

### Implications for research and practice

The findings suggest important avenues for research. In general, research on women’s agency should consider a sequential, mixed-methods approach to scale development and validation to balance contextualization and comparability of scale domains and items. The RAS-17 should be validated over time to confirm its usefulness in panel studies of women’s reproductive agency across pregnancy, delivery, and the post-partum periods, and potential influences on perinatal outcomes. The RAS-17 also may be used to assess the impact of prenatal interventions on women’s reproductive agency, and in turn, on perinatal outcomes [26]. Finally, the RAS-17 should be tested in other pregnant samples (e.g., pregnant women less than 18 years), at other stages of reproduction (e.g., pre-conception and post-partum), and at other reproductive lifecycle stages (menarchy, menopause) to validate the scale across the full reproductive life cycle.

The findings also have implications for practice. Comparability of the RAS-17 across Qatari and non-Qatari Arab women confirms its utility to screen for low reproductive agency among pregnant Arab women in Qatar. Comparability of the RAS-17 also suggests its potential as a screening tool in the sending countries of expatriate Arab women living in Qatar.

## Conclusions

The Reproductive Agency Scale 17 (RAS-17) is a valid, contextual measure of women’s multidimensional reproductive agency that applies to diverse pregnant Arab women of reproductive age. With validation in other samples, the RAS-17 could be used to monitor changes in women’s reproductive agency and its effects on maternal and perinatal health across the reproductive lifecycle. The RAS-17 also has promise to screen and identify women with low reproductive agency, who may be at higher risk for poor perinatal- and maternal-health outcomes.

## Supplementary information

**Additional file 1:** **Table S1.** Distribution of 44 original Reproductive Agency Scale items, pregnant women 19–46 years attending prenatal appointments at Hamad Medical Corporation Maternity Hospital, in Doha, Qatar. Distribution of 44 original Reproductive Agency Scale items, pregnant women 19–46 years attending prenatal appointments at Hamad Medical Corporation Maternity Hospital, in Doha, Qatar.

## Data Availability

The research materials and datasets analyzed in the current study are available from the corresponding author on reasonable request.

## Abbreviations

CAPI: Computer-assisted personal interview
CFA: Confirmatory factor analysis
CFI: Comparative fit index
DHS: Demographic and Health Surveys
EFA: Exploratory factor analysis
HMC: Hamad Medical Corporation
PRES: Pregnancy-related empowerment scale
RMSEA: Root Mean Square Error of Approximation
SDG: Sustainable development goals
TLI: Tucker Lewis Index
RAS: Reproductive Agency Scale

## Footnotes

1 Certain definitions of autonomy may apply poorly in societies where some social relationships are restricted but are valued and enabling amidst structural constraints on women’s actions.
