# Supplementary material for: The Reproductive Agency Scale (RAS-17): development and validation in a cross-sectional study of pregnant Qatari and non-Qatari Arab Women
Source: BMC Pregnancy Childbirth. 2020 Sep 1;20:503. doi: 10.1186/s12884-020-03205-2 (PMC7466495; doi:10.1186/s12884-020-03205-2)
Supplement: Supplementary file 1 — Additional file 1: Table S1. Distribution of 44 original Reproductive Agency Scale items, pregnant women 19–46 years attending prenatal appointments at Hamad Medical Corporation Maternity Hospital, in Doha, Qatar. Distribution of 44 original Reproductive Agency Scale items, pregnant women 19–46 years attending prenatal appointments at Hamad Medical Corporation Maternity Hospital, in Doha, Qatar. [file 12884_2020_3205_MOESM1_ESM.docx]

Supplemental Table 1. Distribution of 44 original Reproductive Agency Scale items, pregnant women 19–46 years attending prenatal appointments at Hamad Medical Corporation Maternity Hospital, in Doha, Qatar

|  | Qatari  *N* = 260 | | | | Non-Qatari  *N* = 424 | | | | All Women  *N* = 684 | | | | |
| --- | --- | --- | --- | --- | --- | --- | --- | --- | --- | --- | --- | --- | --- |
|  | *M* | | *SD* | (*n*) | *M* | | *SD* | (*n*) | *M* | *SD* | (*n*) | *Obs. range* | *p* |
| *^a^Self-reliance, emotional control, and awareness of economic rights. Now I’m going read some statements to you. Please tell me how much you agree or disagree with each statement, where 1 is strongly disagree and 5 is strongly agree.* | | | | | | | | | | | | | |
| Every woman should have a university education | 4.37 | *0.05* | | (260) | 4.61 | *0.03* | | (424) | 4.52 | *0.03* | (684) | 1 – 5 | *** |
| A woman is powerful if she works for pay at a job outside the home | 4.12 | *0.06* | | (260) | 4.01 | *0.05* | | (424) | 4.05 | *0.04* | (684) | 1 – 5 |  |
| A woman should be free to sell her own property | 4.17 | *0.05* | | (259) | 3.99 | *0.04* | | (424) | 4.06 | *0.03* | (683) | 1 – 5 | ** |
| Financial independence makes a woman strong | 4.07 | *0.07* | | (258) | 3.95 | *0.05* | | (423) | 3.99 | *0.04* | (681) | 1 – 5 |  |
| Independent of money, working outside the home strengthens a woman's personality | 3.97 | *0.06* | | (258) | 4.17 | *0.04* | | (424) | 4.10 | *0.04* | (682) | 1 – 5 | ** |
| A woman should solve her own problems without involving others | 3.87 | *0.06* | | (259) | 3.61 | *0.05* | | (424) | 3.71 | *0.04* | (683) | 1 – 5 | ** |
| A woman should make the final decision about the person she will marry | 2.95 | *0.07* | | (259) | 2.85 | *0.06* | | (423) | 2.89 | *0.04* | (682) | 1 – 5 |  |
| A woman becomes stronger when she does not need a man in her life | 2.69 | *0.06* | | (259) | 2.48 | *0.04* | | (423) | 2.56 | *0.04* | (682) | 1 – 5 | ** |
| A married woman should not depend on her family for everything in her life | 3.82 | *0.06* | | (259) | 3.79 | 0.05 | | (423) | 3.80 | 0.04 | (682) | 1 – 5 |  |
| A woman should not let her emotions control her | 4.02 | *0.05* | | (259) | 3.94 | 0.04 | | (423) | 3.97 | 0.03 | (682) | 1 – 5 |  |
| A strong woman will endure difficulties in her life in silence | 3.62 | *0.07* | | (258) | 3.70 | 0.05 | | (423) | 3.67 | 0.04 | (681) | 1 – 5 |  |
| A woman has a weaker personality when she is pregnant | 3.19 | *0.08* | | (258) | 3.08 | 0.06 | | (423) | 3.12 | 0.05 | (681) | 1 – 5 |  |
| A woman has the right to disagree with her husband in decisions related to having children | 3.66 | *0.06* | | (258) | 3.82 | 0.04 | | (422) | 3.76 | 0.03 | (680) | 1 – 5 | * |
| A wife who has children is more valued in society | 2.91 | *0.07* | | (258) | 2.85 | 0.06 | | (422) | 2.87 | 0.04 | (680) | 1 – 5 |  |
| A wife who has no children would not be respected by her family | 1.94 | *0.05* | | (258) | 1.91 | 0.04 | | (422) | 1.92 | 0.03 | (680) | 1 – 5 |  |
| It is better for a wife to stay quiet than to disagree with her mother-in-law | 3.77 | *0.06* | | (257) | 3.41 | 0.05 | | (422) | 3.54 | 0.04 | (679) | 1 – 5 | *** |
| A woman who takes action on her decisions should be admired | 3.55 | *0.06* | | (257) | 3.63 | 0.04 | | (422) | 3.60 | 0.03 | (679) | 1 – 5 |  |
| An unmarried woman should make decisions independently of her parents | 2.31 | *0.06* | | (257) | 2.36 | 0.04 | | (422) | 2.34 | 0.03 | (679) | 1 – 5 |  |
| A woman who does not make her own decisions is a weak woman | 3.10 | *0.07* | | (256) | 3.10 | 0.05 | | (422) | 3.10 | 0.04 | (678) | 1 – 5 |  |
| *^b^Behavioral decision-making. How much influence have you had in the following decisions: 1=no influence, 2=a little influence, 3=some influence, 4=a lot of influence, and 5=I decided by myself.* | | | | | | | | | | | | | |
| Who you married | 4.00 | *0.07* | | (254) | 4.16 | *0.05* | | (421) | 4.10 | *0.04* | (675) | 1 – 5 |  |
| At what age you married | 3.56 | *0.11* | | (254) | 3.59 | *0.08* | | (421) | 3.58 | *0.06* | (675) | 1 – 5 |  |
| The number of children you have | 3.22 | *0.12* | | (212) | 2.98 | *0.08* | | (384) | 3.07 | *0.07* | (596) | 1 – 5 |  |
| When you become pregnant | 2.49 | *0.10* | | (234) | 2.55 | *0.08* | | (397) | 2.53 | *0.06* | (631) | 1 – 5 |  |
| Whether or not you work for pay outside the home | 4.39 | *0.08* | | (204) | 4.02 | *0.07* | | (309) | 4.16 | *0.06* | (513) | 1 – 5 | *** |
| How to spend your own money | 4.56 | *0.07* | | (244) | 4.26 | *0.06* | | (388) | 4.38 | *0.05* | (632) | 1 – 5 | * |
| Which doctor you see for your current pregnancy | 3.49 | *0.11* | | (254) | 2.84 | *0.09* | | (419) | 3.08 | *0.07* | (673) | 1 – 5 | *** |
| When you visit your family | 4.46 | *0.07* | | (248) | 3.98 | *0.07* | | (342) | 4.18 | *0.05* | (590) | 1 – 5 | *** |
| Whether or not you drive | 3.35 | *0.13* | | (157) | 3.93 | *0.09* | | (272) | 3.72 | *0.08* | (429) | 1 – 5 | *** |
| Whether or not you can leave the house unaccompanied | 3.63 | *0.09* | | (240) | 3.55 | *0.07* | | (398) | 3.58 | *0.06* | (638) | 1 – 5 |  |
| What food available in the house you can eat | 4.41 | *0.07* | | (254) | *4.21* | 0.06 | | (419) | 4.28 | *0.04* | (673) | 1 – 5 | * |
| How much you work during your current pregnancy | 4.18 | *0.10* | | (182) | *4.02* | 0.09 | | (217) | 4.10 | *0.07* | (399) | 1 – 5 |  |
| How much you rest during your current pregnancy | 4.45 | *0.06* | | (251) | *4.18* | 0.06 | | (397) | 4.28 | *0.05* | (648) | 1 – 5 | ** |
| Taking medication, vitamins or supplements for yourself | 4.08 | *0.08* | | (252) | *3.85* | 0.07 | | (419) | 3.94 | *0.05* | (671) | 1 – 5 | * |

Supplemental Table 1 (continued)

|  | Qatari  *N* = 260 | | | | Non-Qatari  *N* = 424 | | | | All Women  N = 684 | | | | |
| --- | --- | --- | --- | --- | --- | --- | --- | --- | --- | --- | --- | --- | --- |
|  | Mode | | *%* | (*n*) | Mode | | *%* | (*n*) | Mode | *%* | (*n*) | Obs. range |  |
| *^b^Behavioral freedom of movement. Under what circumstances are you allowed to go to the following places: 1= you are not allowed to go, 2=you do not go, 3=you go with permission if accompanied, 4=you go with permission by yourself, 5=you go without permission if accompanied, and 6=you go without permission by yourself.* | | | | | | | | | | | | | |
| The hospital | 4 | *55.4* | | (251) | 4 | *50.6* | | (419) | 4 | *52.4* | (670) | 1 – 6 |  |
| The movies | 3 | *60.4* | | (235) | 3 | *56.4* | | (388) | 3 | *86.5* | (623) | 1 – 6 |  |
| Events at hotels | 3 | *47.8* | | (226) | 3 | *52.7* | | (383) | 3 | *50.9* | (609) | 1 – 6 |  |
| Restaurants at hotels | 3 | *74.3* | | (237) | 3 | *73.0* | | (400) | 3 | *73.5* | (637) | 1 – 6 |  |
| Coffee shops | 3 | *69.0* | | (242) | 3 | *70.0* | | (404) | 3 | *69.7* | (646) | 1 – 6 |  |
| The mall | 3 | *58.8* | | (250) | 3 | *61.5* | | (418) | 3 | *60.5* | (668) | 1 – 6 |  |
| A female friend’s house | 4 | *71.0* | | (248) | 4 | *72.5* | | (396) | 4 | *71.9* | (644) | 1 – 6 |  |
| Your parent’s home | 4 | *54.6* | | (242) | 4 | *58.1* | | (217) | 4 | *56.2* | (459) | 3 – 6 |  |
| Your husband’s parents’ home | 4 | *39.1* | | (197) | 4 | *44.4* | | (216) | 4 | *41.9* | (413) | 2 – 6 |  |
| Another country | 3 | *94.8* | | (251) | 3 | *70.8* | | (419) | 3 | *79.9* | (670) | 1 – 5 |  |
| Parks or gardens | 3 | *71.7* | | (251) | 3 | *73.0* | | (418) | 3 | *72.5* | (669) | 1 – 6 |  |

Notes. Bolded text indicates item retained in final instrument. Eight items had missing cases at >10%. High percent missing for some variables is due to the question being non-applicable for some women, e.g. not all women in Qatar drive. Missing cases for remaining items ranged 0% – 14% for all women.

^a^Items derived directly from the qualitative transcripts.

^b^Items adapted from existing survey modules, after triangulation with the qualitative data.

*p < 0.05, **p < 0.01, ***p < 0.001 for independent samples t-test (two-tailed).
